# Growth hormone receptor gene influences mitochondrial function and chicken lipid metabolism by AMPK-PGC1α-PPAR signaling pathway

**DOI:** 10.1186/s12864-021-08268-9

**Published:** 2022-03-19

**Authors:** Minmin Yang, Bowen Hu, Donglei Sun, Changbin Zhao, Haohui Wei, Dajian Li, Zhiying Liao, Yongxia Zhao, Jinping Liang, Meiqing Shi, Qingbin Luo, Qinghua Nie, Xiquan Zhang, Dexiang Zhang, Hongmei Li

**Affiliations:** 1 State Key Laboratory for Conservation and Utilization of Subtropical Agro-bioresources, College of Animal Science, South China Agricultural University, Guangzhou, 510642 Guangdong China; 2 Guangdong Provincial Key Lab of AgroAnimal Genomics and Molecular Breeding and Key Lab of Chicken Genetics, Breeding and Reproduction, Ministry of Agriculture, Guangzhou, 510642 Guangdong China; 3 Division of Immunology, Virginia-Maryland Regional College of Veterinary Medicine, University of Maryland, College Park, MD USA

**Keywords:** *GHR*, Lipid metabolism, Mitochondrial function, Pre-adipocyte differentiation, Sex-linked dwarf chickens

## Abstract

**Background:**

Adipose tissue is an important endocrine and energy-storage organ in organisms, and it plays a crucial role in the energy-metabolism balance. Previous studies have found that sex-linked dwarf (SLD) chickens generally have excessively high abdominal fat deposition during the growing period, which increases feeding costs. However, the underlying mechanism of this fat deposition during the growth of SLD chickens remains unknown.

**Results:**

The Oil Red O staining showed that the lipid-droplet area of SLD chickens was larger than that of normal chickens in E15 and 14d. Consistently, TG content in the livers of SLD chickens was higher than that of normal chickens in E15 and 14d. Further, lower ΔΨm and lower ATP levels and higher MDA levels were observed in SLD chickens than normal chickens in both E15 and 14d. We also found that overexpression of *GHR* reduced the expression of genes related to lipid metabolism (*AMPK*, *PGC1α*, *PPARγ*, *FAS*, *C/EBP*) and oxidative phosphorylation (*CYTB*, *CYTC*, *COX1*, *ATP*), as well as reducing ΔΨm and ATP levels and increasing MDA levels. In addition, overexpression of *GHR* inhibited fat deposition in CPPAs, as measured by Oil Red O staining. On the contrary, knockdown of *GHR* had the opposite effects in vitro.

**Conclusions:**

In summary, we demonstrate that *GHR* promotes mitochondrial function and inhibits lipid peroxidation as well as fat deposition in vivo and in vitro. Therefore, *GHR* is essential for maintaining the stability of lipid metabolism and regulating mitochondrial function in chicken.

**Supplementary Information:**

The online version contains supplementary material available at 10.1186/s12864-021-08268-9.

## Background

Adipose tissue is an important endocrine and energy-storage organ in organisms, and it plays a crucial role in the energy-metabolism balance of organisms. In poultry, the liver is the main site of fat synthesis, synthesizing about 90% of body fat, and then transferred to adipose tissue [1]. Triglycerides (TGs) are the main form of fat, and the relative speed of the synthesis and decomposition of TG determines the deposition or depletion of fat. Lipid metabolism is complex process, and it includes the synthesis of lipids, the decomposition of fatty acids, and the transport of intermediate products [2, 3]. The three processes of lipid metabolism are closely related, and any alternation in any of these affects the balance of lipid levels in the individual [4]. Furthermore, animal adipocytes originate from mesoderm cells after undergoing stages such as mesenchymal stem cells, pre-adipocytes, and pre-differentiated adipocytes, eventually differentiating into mature adipocytes. In this series of processes, transcription factors related to lipid metabolism play a significant part in regulation in coordination with each other, such as PPARγ and C/EBPs. Previous studies have confirmed that PPARγ is the main regulator of adipogenesis, and in the absence of PPARγ, no transcription factor has been found that can initiate adipocyte differentiation into mature adipocytes [5, 6]. C/EBPs are also a necessary factor for adipocyte differentiation, and C/EBPα is highly expressed in the early stages of adipocyte differentiation [7]. In cooperation with C/EBP transcription factor, PPARγ can affect fatty acid storage in adipose tissue and induce mature adipocytes from pre-adipocytes, which is indispensable in adipocyte differentiation.

Mitochondria are the main sites of fatty acid oxidation and provide precursors for intracellular lipid anabolism. Thus, they are closely related to lipid metabolism. In addition to the accumulation of TGs, differentiated adipocytes are also accompanied by mitochondrial proliferations, and the stability of the mitochondrial function is essential for the body to maintain normal lipid levels. Under physiological conditions, mitochondrial oxidative phosphorylation (OXPHOS) produces free radicals and antioxidant levels to maintain a dynamic balance. Abnormal lipid metabolism causes mitochondria to produce excessive reactive oxygen species, which further breaks the balance and leads to mitochondrial lipid peroxidation, thereby destroying the mitochondrial membrane potential (ΔΨm), leading to a decrease in adenosine triphosphate (ATP) production [8, 9]. In addition, these changes in mitochondria exacerbate lipid peroxidation and the production of reactive oxygen species. Previous studies have found that patients with symptoms of obesity or fatty liver have damaged mitochondrial structures and lowered liver ATP levels [10]. Similarly, increased liver TG contents, higher active oxygen levels, and lower ATP levels have been observed in obese mice [11]. Here, AMPK plays an important role in regulating the energy balance of animal cells. When the production of ATP decreases, AMPK is activated [12]. Previous study has shown that AMPK can promote the formation of mitochondria, for which peroxisome proliferator-activated receptor γ co-activator 1α (PGC1α) is the key intermediate link, and AMPK directly or indirectly activates PGC1α, thereby promoting mitochondrial biogenesis [13]. Therefore, *AMPK*, as an upstream regulatory gene for PGC1α, has regulatory significance for mitochondrial biogenesis, and it also promotes fat oxidation metabolism at the same time.

The fat deposition of chickens is mainly manifested in subcutaneous and abdominal fat [14, 15]. While increases in the weight of chickens is desirable for the poultry industry, it is accompanied by the deposition of excessive fat, which causes unnecessary feed waste and reduces economic efficiency [16]. Sex-linked dwarf (SLD) chickens, which are caused by mutations in the growth hormone receptor (*GHR*) gene, are feasible models for understanding the role of *GHR* in poultry [17]. Previous studies have found that SLD chickens generally have excessively high abdominal fat deposition during the growth period, which increases feeding costs and is associated with Runting and Stunting Syndrome chickens [18, 19]. However, the underlying mechanisms of fat deposition during the growth of SLD chickens remains unknown.

Based on these findings above, we used SLD and normal chicken livers for our research object, and overexpressed or interfered with in chicken primary hepatocytes (CPHs) and primary pre-adipocytes (CPPAs), to investigate the effects of the *GHR* on lipid metabolism and mitochondrial function in vivo and in vitro, respectively. This study elucidates the underlying mechanisms of fat deposition during the growth of SLD chickens, which may provide a reference for human metabolic diseases.

## Results

### Oil red O staining of SLD chickens and normal chickens

**Fig. 1:**
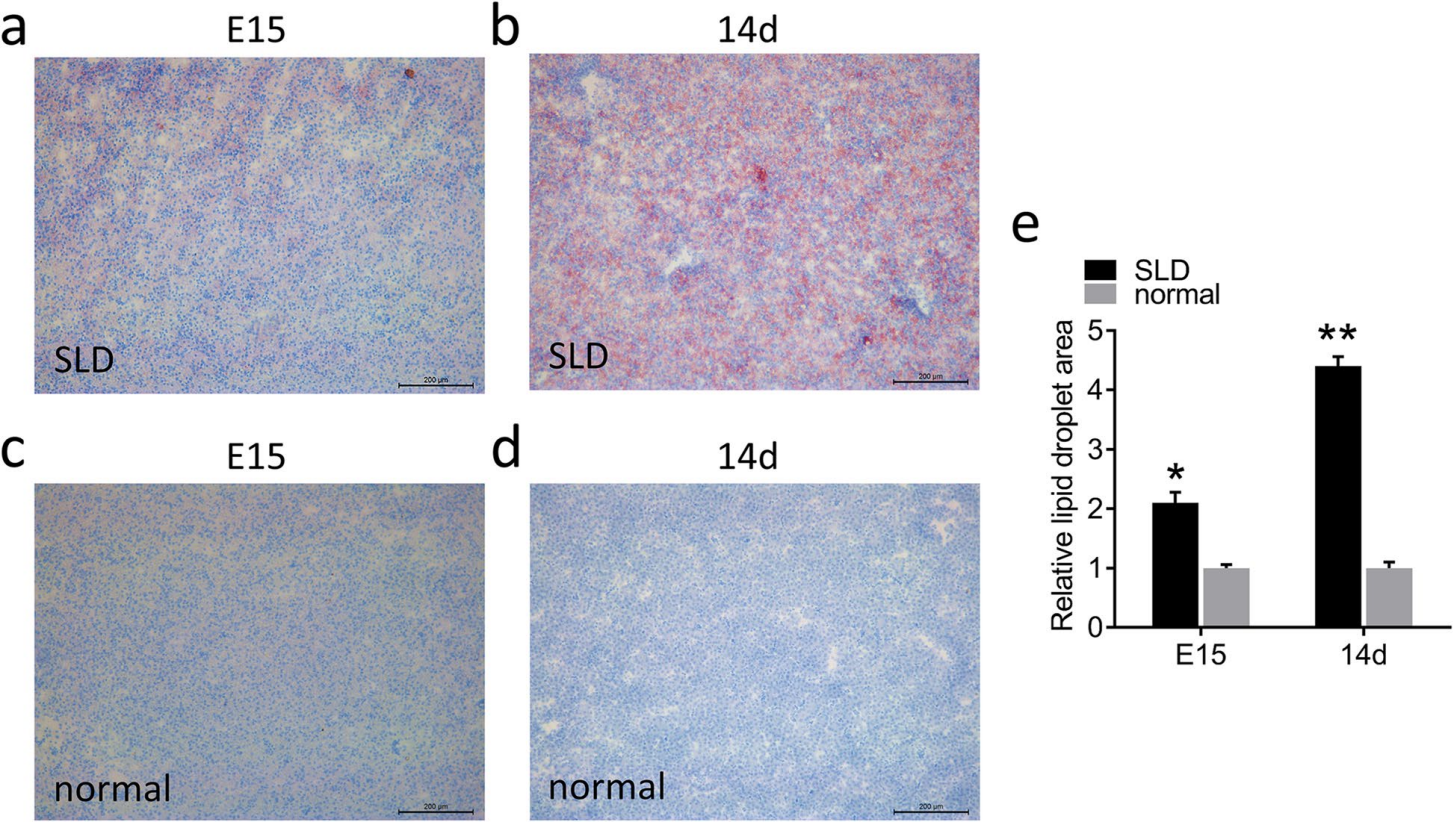
Oil Red O staining of SLD chickens and normal chickens. **a** Oil Red O staining of liver sections of E15 SLD chickens; bar, 200 μm. **b** Oil Red O staining of liver sections of 14d SLD chickens; bar, 200 μm. **c** Oil Red O staining of liver sections of E15 normal chickens; bar, 200 μm. **d** Oil Red O staining of liver sections of 14d normal chickens; bar, 200 μm. **e** Analyzed the area of lipid droplets in SLD chickens and normal chickens. Data are expressed as means ± SEMs, *P < 0.05, **P < 0.01, ***P < 0.001; *n* = 6

First, we performed Oil Red O staining to explore the effects of *GHR* on the accumulation of lipid droplets in the liver tissue of SLD and normal chickens. Lipid droplets exist in the form of TGs in tissues, and Oil Red O specifically stains TGs in liver tissues to red. The results showed that E15 SLD chickens had obvious lipid droplets, which was further exacerbated at 14d (Fig. 1a, b). However, normal chickens showed no significant change after Oil Red O staining, which only exhibited a few lipid droplets (Fig. 1c, d). Next, we analyzed the area of lipid droplets in SLD and normal chickens. It was shown that the liver lipid droplet area of the SLD chickens was significantly increased from that of normal chickens in both E15 and 14d (Fig. 1e).

### Detection of liver mitochondrial function in SLD and normal chickens

**Fig. 2:**
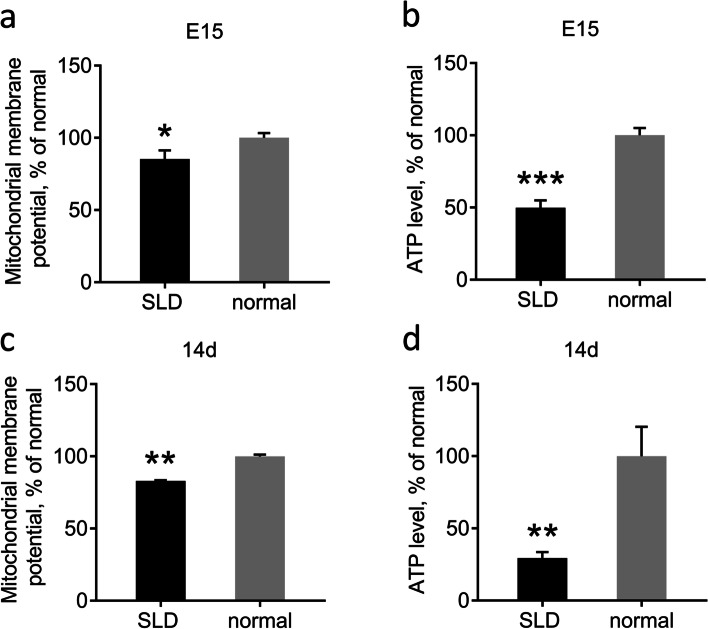
Detection of liver mitochondrial function in SLD chickens and normal chickens. **a** Detection of liver ΔΨm in E15 SLD chickens and normal chickens. **b** Detection of liver ATP level in E15 SLD chickens and normal chickens. **c** Detection of liver ΔΨm in 14d SLD chickens and normal chickens. **d** Detection of liver ATP levels in 14d SLD chickens and normal chickens. Data are expressed as means ± SEMs, *P < 0.05, **P < 0.01, ***P < 0.001; n = 6

We next measured mitochondrial function in the livers of SLD chickens and normal chickens at E15 and 14d to understand whether *GHR* affected the function of mitochondria in the liver during the early growth stage. The results showed that liver ΔΨm for the SLD chickens was 15% lower than that for normal chickens at E15 and 12% lower at 14d (Fig. 2a, c). Liver ATP levels for the SLD chickens were 51% lower than those for normal chickens in E15 and 70% lower than in 14d (Fig. 2b, d). These results reveal that mitochondrial function is impaired in SLD chicken livers in E15 and 14d.

### Detection of liver lipid levels in SLD and normal chickens

**Fig. 3:**
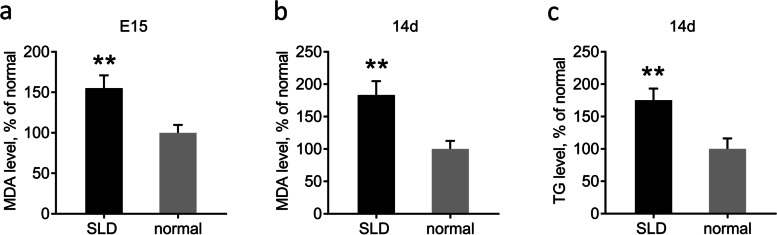
Detection of liver lipid levels in SLD chickens and normal chickens. **a** Detection of liver MDA levels in E15 SLD chickens and normal chickens. **b** Detection of liver MDA levels in 14d SLD chickens and normal chickens. **c** Detection of liver TG level in 14d SLD chickens and normal chickens. Data are expressed as means ± SEMs, *P < 0.05, **P < 0.01, ***P < 0.001; *n* = 6

To investigate lipid levels, we measured the MDA and TG levels in E15 and 14d SLD and normal chicken livers. The liver MDA levels in the SLD chickens were 55% higher than those in the normal chickens at E15 and 83% lower at 14d (Fig. 3a, b). Further, liver TG levels in the SLD chickens were 55% higher than those in the normal chickens in 14d (Fig. 3c). These results reveal lipid peroxidation and increased TG contents, indicating abnormal lipid levels in the SLD chicken livers.

### Detection of chicken serum protein and enzyme activity by ELISA

**Fig. 4:**
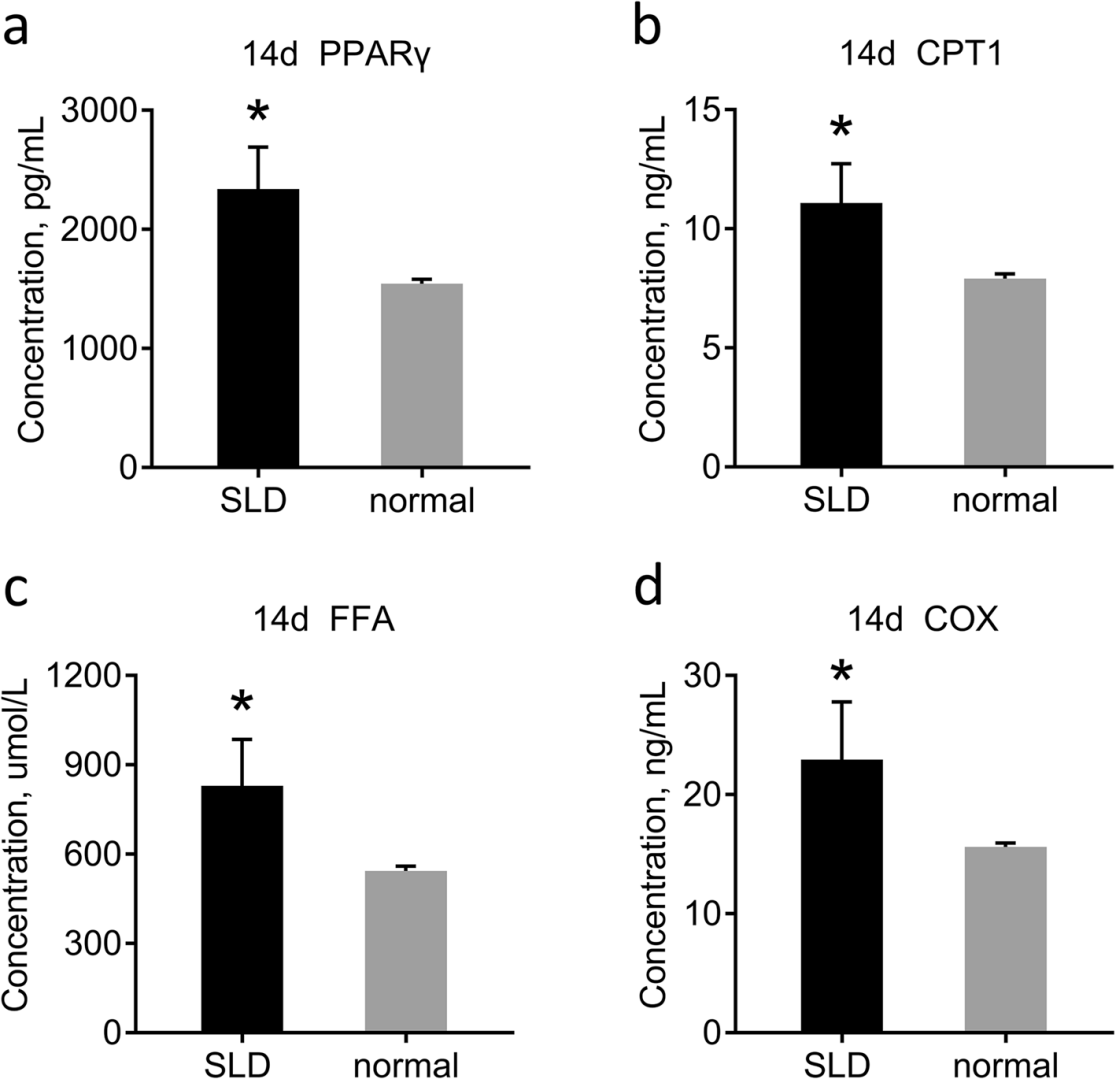
Detection of chicken serum protein and enzyme activity by ELISA. **a** Detection of concentrations of PPARγ in serum of 14d SLD chickens and normal chickens. **b** Detection of concentrations of CPT1 in serum of 14d SLD chickens and normal chickens. **c** Detection of concentrations of FFA in serum of 14d SLD chickens and normal chickens. **d** Detection of concentrations of COX in serum of 14d SLD chickens and normal chickens. Data are expressed as means ± SEMs, *P < 0.05, **P < 0.01, ***P < 0.001; n = 6

Protein levels and enzyme activities in animal serum reflect metabolic levels in the body. Therefore, we analyzed the content of various proteins in the serum of 14d SLD and normal chickens. The concentrations of PPARγ, CPT1, FFA, and COX in the serum of 14d SLD chickens were all greater than those of normal chickens, indicating that lipid metabolism is active in SLD chickens (Fig. 4a–d). This might be associated with mitochondrial dysfunction in SLD chicken livers.

### Overexpression and knockdown of *GHR* gene in vitro

**Fig. 5:**
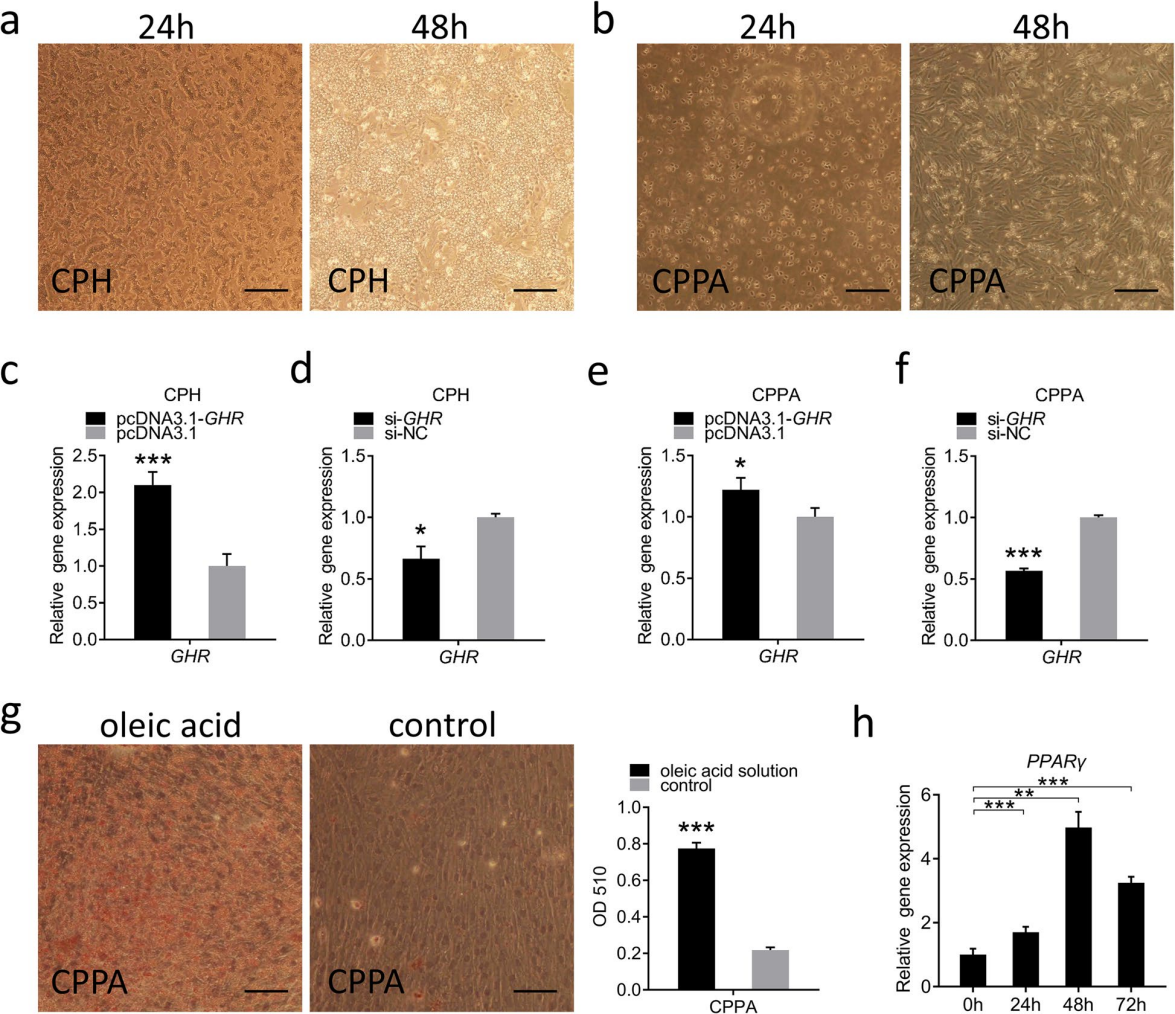
Overexpression and knockdown of *GHR* gene in vitro. **a** Morphology of CPHs at 24 h and 48 h; bar 100 μm. **b** Morphology of the CPPAs at 24 h and 48 h; bar 100 μm. **c, d** Transfection efficiency was measured using qRT-PCR in CPHs. **e, f** Transfection efficiency was measured using qRT-PCR in CPPAs. **g** Oil Red O staining in CPPAs after induction by oleic acid-induced differentiation solution for 2 days; bar 100 μm. **h** Spatiotemporal expression of *PPARγ* gene during differentiation of CPPAs. Data are expressed as means ± SEMs, *P < 0.05, **P < 0.01, ***P < 0.001; *n* = 6

To explore the role of *GHR* in vitro, we interfered with the expression of *GHR* in CPHs and CPPAs (Fig. 5a, b). Transfection efficiency was measured using qRT-PCR and the fluorescence of pcDNA3.1-EGFP and siNC-Cy3 (Fig. S1). The expression of the *GHR* gene was significantly up-, and downregulated in *GHR* overexpression and knockdown cells, respectively (Fig. 5c–f), indicating that we had successfully overexpressed or interfered with the expression of the gene in CPHs and CPPAs. Further, we performed Oil Red O staining in CPPAs after induction with oleic acid differentiation solution for 2 days. The OD value of the cells in the oleic acid-induced differentiation group, which represents lipid accumulation, was significantly higher than that of the control group (Fig. 5g). In addition, the *PPARγ* gene induces CPPAs to differentiate into mature adipocytes, and it is a marker gene for fat differentiation. Next, we measured the expression of *PPARγ* in CPPAs at 0 h, 24 h, 48 h, and 72 h with oleic acid-induced differentiation solution. The results showed that *PPARγ* expression increased with increase in induction differentiation time and reached a higher expression level at 48 h (Fig. 5h), indicating that we had successfully isolated the CPPAs.

### Effects of *GHR* gene on the expression of lipid metabolism and OXPHOS-related genes in vitro

**Fig. 6:**
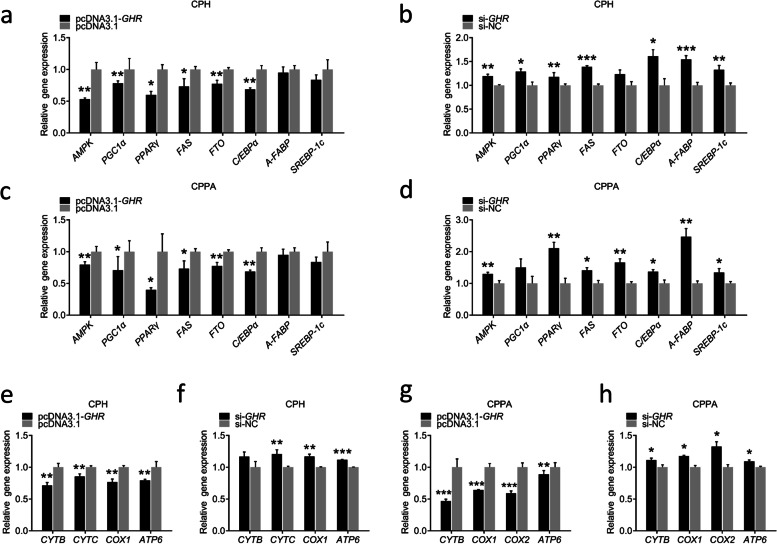
Effects of *GHR* gene on the expression of lipid metabolism and OXPHOS-related genes in vitro. **a** Expression of genes related to lipid metabolism in CPHs at 48 h after transfection with pcDNA3.1-*GHR* and pcDNA3.1. **b** Expression of genes related to lipid metabolism in CPHs at 48 h after transfection with si-*GHR* and si-NC. **c** Expression of genes related to lipid metabolism in CPPAs at 48 h after transfection with pcDNA3.1-*GHR* and pcDNA3.1. **d** Expression of genes related to lipid metabolism in CPPAs at 48 h after transfection with si-*GHR* and si-NC. **e** Expression of OXPHOS-related genes in CPHs at 48 h after transfection with pcDNA3.1-*GHR* and pcDNA3.1. **f** Expression of OXPHOS-related genes in CPHs at 48 h after transfection with si-*GHR* and si-NC. **g** Expression of OXPHOS-related genes in CPPAs at 48 h after transfection with pcDNA3.1-*GHR* and pcDNA3.1. **h** Expression of OXPHOS-related genes in CPPAs at 48 h after transfection with si-*GHR* and si-NC. Data are expressed as means ± SEMs, *P < 0.05, **P < 0.01, ***P < 0.001; *n* = 6

Next, we explored the role of *GHR* on the expression of lipid metabolism and OXPHOS-related genes in CPHs and CPPAs. It was found that the overexpression of *GHR* reduced the expression of genes related to lipid metabolism (*AMPK*, *PGC1α*, *PPARγ*, *FAS*, *FTO*, *C/EBP*, *A-FABP*, *SREBP-1c*), and knockdown of *GHR* increased the expression of these genes (Fig. 6a, b). The same observations were made in CPPAs (Fig. 6c, d), indicating that *GHR* might inhibit lipid metabolism via AMPK signaling in vitro. Furthermore, we found that the overexpression of *GHR* reduced the expression of OXPHOS-related genes (*CYTB*, *CYTC*, *COX1*, *ATP*), whereas knockdown of *GHR* increased the expression of these genes (Fig. 6e, f). The same results were observed in CPPAs (Fig. 6g, h), indicating that *GHR* inhibits the transcription of OXPHOS-related genes in vitro.

### Effects of *GHR* on lipid levels in vitro

**Fig. 7:**
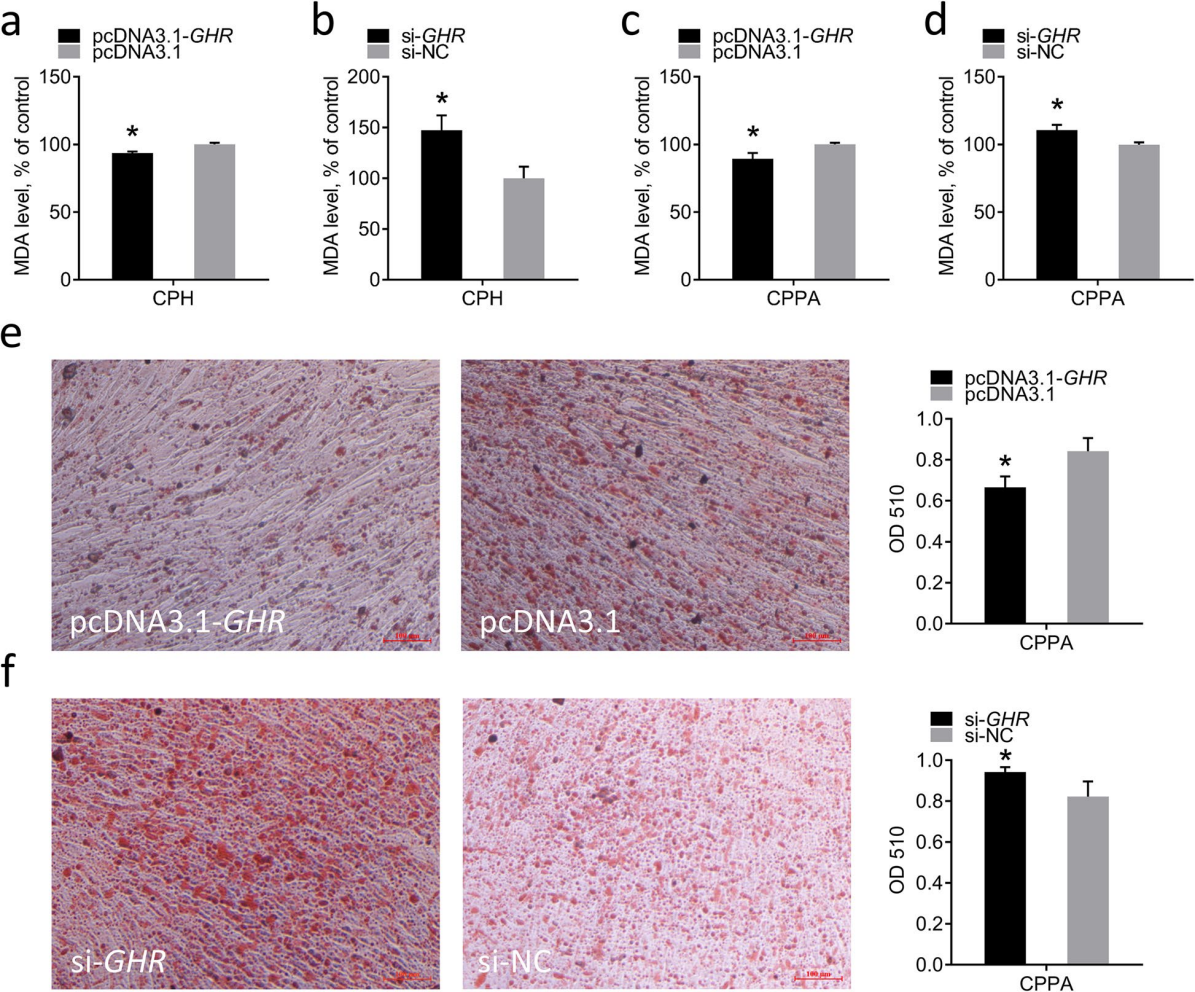
Effects of *GHR* on lipid level in vitro. **a** Detection of MDA levels in CPHs at 48 h after transfection with pcDNA3.1-*GHR* and pcDNA3.1. **b** Detection of MDA level in CPHs at 48 h after transfection with si-*GHR* and si-NC. **c** Detection of MDA level in CPPAs at 48 h after transfection with pcDNA3.1-*GHR* and pcDNA3.1. **d** Detection of MDA level in CPPAs at 48 h after transfection with si-*GHR* and si-NC. **e** Oil Red O staining of CPPAs induced by oleic acid differentiation solution at 48 h after transfection with pcDNA3.1-*GHR* and pcDNA3.1; bar 100 μm. **f** Oil Red O staining of CPPAs induced by oleic acid differentiation solution at 48 h after transfection with si-*GHR* and si-NC; bar 100 μm. Data are expressed as means ± SEMs, *P < 0.05, **P < 0.01, ***P < 0.001; n = 6

To investigate the effects of *GHR* on lipid levels in vitro, we measured MDA levels in CPHs and CPPAs and performed Oil Red O staining of CPPAs induced by oleic acid differentiation. The results showed that overexpression of *GHR* reduced MDA levels, whereas knockdown of *GHR* increased MDA levels in CPHs (Fig. 7a, b). The same results were observed in CPPAs (Fig. 7c, d), indicating that *GHR* inhibited lipid peroxidation in vitro. We performed Oil Red O staining of CPPAs induced by oleic acid differentiation solution at 48 h after transfection. It was found that overexpression of *GHR* significantly reduced OD values of cells, whereas knockdown of *GHR* significantly increased OD values of cells in CPPAs (Fig. 7e, f), indicating that *GHR* inhibits fat deposition in vitro.

### Effects of *GHR* on mitochondrial function in vitro

**Fig. 8:**
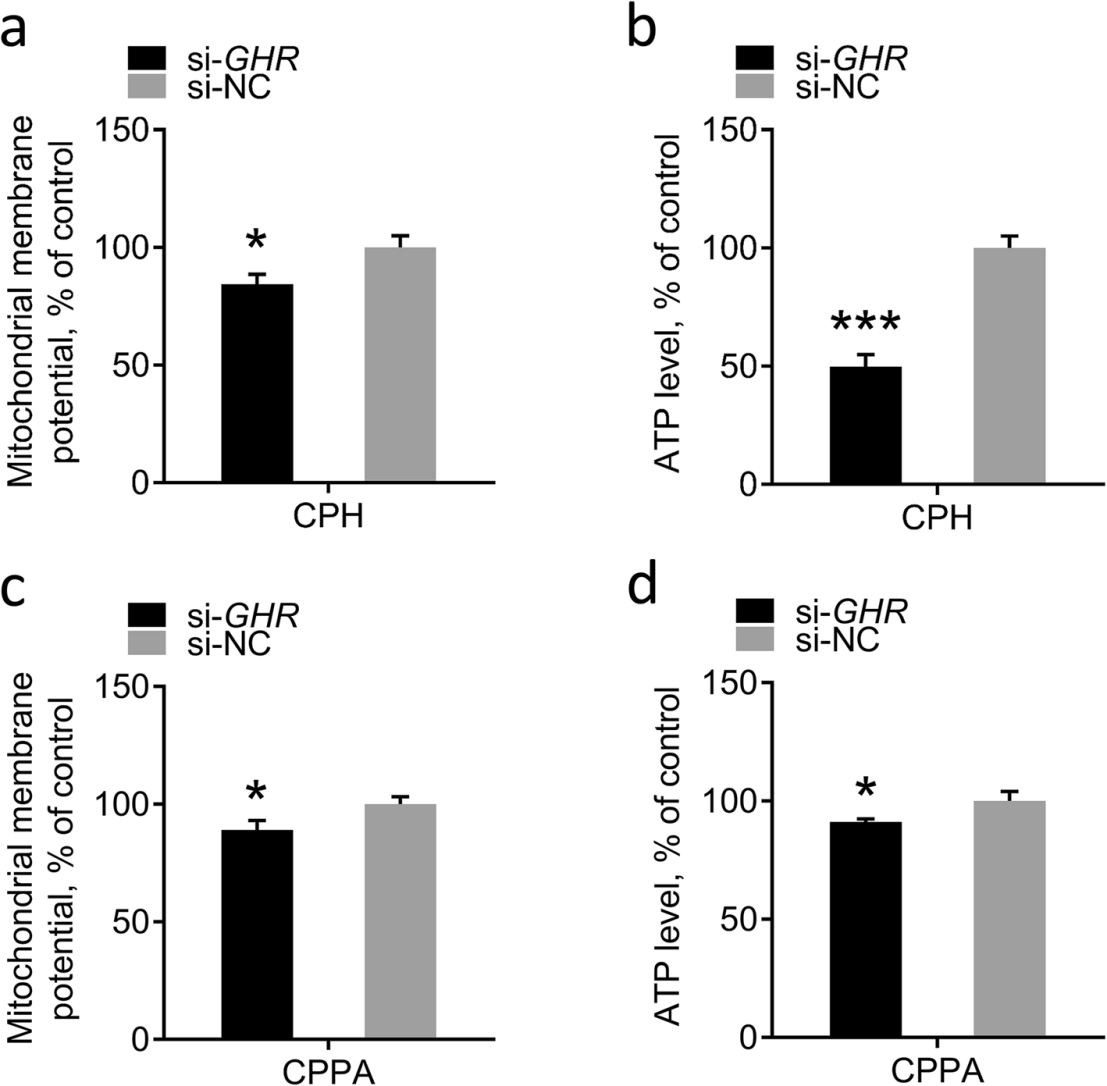
Effects of *GHR* gene on mitochondrial function in vitro. **a** Detection of ΔΨm in CPHs at 48 h after transfection with si-*GHR* and si-NC. **b** Detection of ATP level in CPHs at 48 h after transfection with si-*GHR* and si-NC. **c** Detection of ΔΨm in CPPAs at 48 h after transfection with si-*GHR* and si-NC. **d** Detection of ATP levels in CPPAs at 48 h after transfection with si-*GHR* and si-NC. Data are expressed as means ± SEMs, *P < 0.05, **P < 0.01, ***P < 0.001; n = 6

To further understand the effects of *GHR* on mitochondrial function in vitro, we measured ΔΨm and ATP levels in CPHs and CPPAs. The results showed that the ΔΨm of the *GHR* knockdown group was lower than that in the control groups in CPHs, but the significance was not obvious (Fig. 8a). Consistently with this, the ATP levels of the *GHR* knockdown groups were significantly reduced compared to the control groups in CPHs (Fig. 8b). The same results were observed in CPPAs (Fig. 8c, d), indicating that mitochondrial function is impaired in *GHR* knockdown cells.

## Discussion

Adipose tissue is a main component of the body. Uniform distribution and content of fat provide energy and ensure healthy growth in poultry. Fat is an important indicator of meat quality, and the degree of fat deposition has an important impact on the quality of the carcass and meat [20]. Excessive fat deposition reduces feed utilization and causes economic losses in poultry. Consumers who ingest more animal fat may have reduced health status, which is harmful to the body of the poultry, causing various diseases. Defects in mitochondria, the final place where various substances oxidize and release energy, directly affect the normal function of adipose tissue, which leads to imbalances of energy throughout the body. The liver is the main fat-producing part of poultry, and it can be studied during the embryonic period [21]. This study focuses on lipid metabolism and mitochondrial function in the liver and adipose tissue of poultry.

First, we used SLD and normal chickens as research objects to measure the mitochondrial function and lipid levels of liver, as well as the activities of related proteins and enzymes in serum. Oil Red O staining is commonly used in liver histopathological detection, as it has a strong binding ability with lipids and can reveal the distribution of lipid droplets and TG content in liver tissues [22]. Here, we found that SLD chicken liver had lipid droplets at E15, but normal chicken liver at E15 had few or no lipid droplets. Under the same feeding conditions, liver lipid droplets of 14d SLD chickens increased relative to those of E15 SLD chickens and 14d normal chickens. Moreover, excessive FFA in serum is excessively deposited in non-adipose tissue in the form of TG and causes tissue damage [23], including in the liver. Consistent with this, increased FFA content was also observed in the serum of SLD chickens. We measured the TG content of liver tissue, which were consistent with those of the Oil Red O staining, indicating that the liver of SLD chickens is more active in lipid synthesis from the embryonic stage. These results support the fact observed in production that SLD chickens are prone to fat deposition.

Impaired mitochondrial function directly affects the function of adipose tissue, which in turn affects energy metabolism in the entire body. The main product of membrane lipid peroxidation is MDA, and MDA contents can reflect the degree of accumulation of lipid peroxides in the body, which can change the ΔΨm. The stability of ΔΨm is a prerequisite for maintaining OXPHOS and generating ATP, which is conducive to the maintenance of normal physiological functions in cells. The reduction of ΔΨm is an irreversible change in early apoptosis [24], and it is generally accompanied by reduced ATP production, which reflects the functional state of mitochondria. Previous study has revealed that intracellular ATP levels are in a fluctuating balance, and increased levels of fat and liver ATP are observed in high-fat fed obese mouse models [25]. Excessive ATP production leads to insulin resistance, sugar metabolism becomes disordered, and various metabolic diseases are induced. In addition, reduced ATP levels may also induce metabolic imbalances. Pathological studies have shown that when mitochondrial dysfunction occurs, glucose and lipid metabolism are weakened, ATP production may be reduced accordingly, and energy deficiencies prevent normal physiological activities. Previous studies have found that ΔΨm, ATP levels and oxygen consumption were all reduced accordingly in *GHRKO* and *GHR* knockdown cells [26, 27]. However, there is evidence that decreased ATP levels may have protective effects on the body in systemic inflammatory response condition [28]. We also found that SLD chicken liver had higher MDA levels and lower ΔΨm and ATP levels than normal chickens, indicating that the mitochondrial function of SLD in the liver of chickens is impaired at E15 and 14d. These results are also concordant with our previous study showing that the skeletal muscle and liver mitochondrial function of SLD chickens are impaired at 7w [18, 27].

Mutations in the *GHR* gene are the cause of SLD chicken, and previous study has also shown that *GHRKO* mice generally show slow growth and high fat [29]. Therefore, we synthesized the interfering fragments of the *GHR* gene and then transfected CPHs and CPPAs to study the effects of *GHR* defects on lipid levels and mitochondrial function in vitro. *PPARγ* and *FAS* are key genes that regulate the differentiation of adipocytes and are also marker genes that prompt CPPAs to differentiate into mature cells [30]. In this study, we found that knockdown of *GHR* up-regulated the expression of genes related to fat differentiation (*PPARγ*, *FAS*, and so forth); overexpression of *GHR* had the opposite effects in vitro. Next, we measured TG content by Oil Red O staining in differentiated CPPAs and revealed that defective *GHR* increased TG content. The increased differentiation of adipocytes can cause their excessive accumulation and eventually lead to fat deposition [2]. Therefore, we argue that *GHR* has a positive effect on the differentiation of CPPAs into mature adipocytes, and *GHR* can inhibit the differentiation of fat, which is consistent with excessive lipid deposition in SLD chickens.

In addition, the overexpression of *GHR* leads to lower expression of OXPHOS-related genes in vitro, accompanied by increased ΔΨm and ATP levels along with reduced MDA levels. Knockdown of *GHR* had the opposite results, indicating that *GHR* promotes mitochondrial function and inhibits lipid peroxidation in vitro. These results above exhibit the same trend in vivo. Overexpression of *GHR* leads to low expression of OXPHOS-related genes in CPHs and CPPAs, whereas knockdown of *GHR* had opposite effects in vitro. However, it was found that the expression of OXPHOS-related genes is down-regulated in *GHR* knockdown of DF-1 cells [27], which may indicate that the effects of *GHR* on mitochondrial biogenesis exhibit a compensatory phenomenon in the early stages of growth and development or has cell-dependent features in vitro.

## Conclusions

In summary, we demonstrate that *GHR* promotes mitochondrial function and inhibits lipid peroxidation and fat deposition in vivo and in vitro. Therefore, *GHR* gene is essential for maintaining the stability of lipid metabolism by regulating mitochondrial function in chicken.

## Methods

### Chickens

For the isolation of CPHs, 15-embryo-age normal chickens (*n* = 20) from the 202 strains were used. For the isolation of CPPAs, 14-day-old normal chickens (n = 20) from the 202 strains were used. Furthermore, the 15-embryo-age and 14-day-old SLD chickens from the 301 strain and normal chickens from the 202 strain (*n* = 6) were sampled as previously described [27]. All chicks were purchased from the WenShi Group Co., Ltd. (Guangdong, China) and were provided with standard poultry feed and fresh water and reared in an optimum temperature range (27–37 °C). The pentobarbital was used for euthanization by intraperitoneal injection at a dose of 40 mg/kg of body weight.

### Oil red O staining

Liver tissues were removed from the chick livers and fixed in 10% neutral formaldehyde. The samples were stained using Oil Red O, and the slices were observed through an optical microscope (DMi8; Leica, Germany) and photographed (LAS X Life Science Microscope Software Platform; Leica, Germany). In addition, cells were seeded in 12-well culture plates. After transfection and differentiation, the differentiated cells were washed in PBS and incubated with 10% neutral formaldehyde for 30 min. Subsequently, differentiated cells were dyed with Oil Red O, and observed with an optical microscope (DMi8; Leica, Germany) and photographed (LAS X Life Science Microscope Software Platform; Leica, Germany). Finally, the cell stain was extracted with isopropanol and analyzed at 510 nm absorbance with a Fluorescence/Multi-Detection Microplate Reader (BioTek, USA).

### Detection of the concentration of serum protein and TG level

The concentrations of PPARγ, CPT1, FFA, and COX in serum were determined with commercial ELISA kits (mlbio, China) according to the manufacturer’s protocol. TG levels in the liver and serum were determined with a TG assay kit (Nanjing Jiangcheng, China) according to the manufacturer’s protocol.

### Quantitative real-time PCR

RNA of tissues and cells was extracted using RNAiso reagent (Takara, Japan) according to the manufacturer’s protocol. RNA concentration and the OD values of 260/280 were detected with a Nanodrop 2000c spectrophotometer (Thermo, USA). RNA was stored at − 80 °C for later use. cDNA was synthesized using a PrimeScript RT Reagent Kit (Takara, Japan) for quantitative real-time PCR (qRT-PCR). We selected MonAmp™ ChemoHS qPCR Mix (Monad Co., LTD, Guangzhou, China) for qRT-PCR measuring by a Bio-Rad CFX96 Real-Time Detection instrument (Bio-Rad, USA) according to the manufacturer’s protocol. Relative gene expression was measured twice for each reaction, and *GADPH* and *β-actin* were used as controls. The primers used for qRT-PCR are listed in Table 1.

**Table 1:** Primer sequences of qRT-PCR

Genes	Primer sequences	Notes
*PPARγ*	F-CTCCTTCTCCTCCCTATTT R-TTTCTTATGGATGCGACA	qRT-PCR
*FAS*	F-CGCAGGCATAGCAGGAAA R-CCAAAGAAGGAGGCATCAA	qRT-PCR
*FTO*	F-AGGGCAAACTTCCATACC R-ACGAGACACTGGGGTAAA	qRT-PCR
*C/EBPα*	F- GACAAGAACAGCAACGAGTACC R-CCTGAAGATGCCCCGCAGAGT	qRT-PCR
*SREBP-1c*	F-GGTCCGGGCCATGTTGA R-CAGGTTGGTGCGGGTGA	qRT-PCR
*A-FABP*	F-ATGTGCGACCAGTTTGT R-TCACCATTGATGCTGATAG	qRT-PCR
*CYTB*	F-CAGCAGACACATCCCTAGCC R-GAAGAATGAGGCGCCGTTTG	qRT-PCR
*COX1*	F-ACTACTTACCGACCGCAACC R-CCGAAACCTGGGAGGATGAG	qRT-PCR
*COX2*	F-TCGGGGTAAAAACAGACGCA R-ACTCCTGGTCGAGTGGTGAT	qRT-PCR
*COX3*	F-CCCAAGCCCATGACCAATCT R-TGGAAGGTGCTTTCTCGGAC	qRT-PCR
*ATP6*	F-TACAGCCACAATCGCCCTAC R-AGGACGAAGACGTAGGCTTG	qRT-PCR
*PGC1α*	F-TCCTTTCCTCAACGCAGGTC R-TCTTGCACGTGAGGGAGAAC	qRT-PCR
*GHR*	F-GCAAGTGCAGGTCACCTGAG R-CCGGACATTCTTTCCAGTCT	qRT-PCR
*GAPDH*	F-CAACTTTGGCATTGTGGAGG R-CGCTGGGATGATGTTCTGG	qRT-PCR
*β-actin*	F-GATATTGCTGCGCTCGTTG R-TTCAGGGTCAGGATACCTCTTT	qRT-PCR

### Extraction of chicken primary hepatocytes and chicken primary pre-adipocytes

CPHs were isolated from the liver of 15-embryo-age chickens according to the protocol as previously described [31]. In brief, 20 eggs of 15 embryonic age were taken and wiped with 75% alcohol for disinfection. Tweezers were used to grab the liver, and put it into D-hank’s solution preheated to 37 °C. Washed liver sample several times to remove blood and impurities, and digested them in a 37 °C incubator for 30 min with 2.5 mg/ml type IV collagenase solution. Then, filtered the digested liver mixture with 200 mesh and 400 mesh sieves, centrifuged at 1000 r/min for 5 min. After the centrifugation, discarded the supernatant and re-suspended it in Williams’E medium containing 15% fetal bovine serum. Slowly added the cell suspension dropwise to the centrifuge tube equipped with percoll centrifugal solution, and centrifuged at 3000 r/min for 15 min. After obtained the CPHs, the serum-containing Williams’E medium was added to re-suspend the cells.

CPPAs were isolated from the abdominal fat of 14-day-old chickens, and the differentiation of CPPAs were induced by oleic acid differentiation solution according to the protocol as previously described [30]. In brief, 14-day-old chickens were immersed in 75% alcohol for disinfection for 10 min. Then, the abdominal fat was removed in a sterile operating table as completely as possible, and washed with DMEM/F12 medium several times to remove blood vessels and other impurities. After obtaining the adipose tissue, added 2 mg/mL type I collagenase, and incubated for 30 min in a 37 °C incubator. After digestion, CPPAs were filtered with a 200-mesh sieve and centrifuged at 1300 r/min for 5 min. After obtained the CPPAs, the serum-containing DMEM/F12 medium was added to re-suspend the cells.

### Cell culture

CPHs and CPPAs were cultured in William’s E Medium (Gibco, USA) and DMEM/F12 Medium (Gibco, USA) with 15% fetal bovine serum (ExCell Bio, China) and 0.2% penicillin/streptomycin (Invitrogen, USA), respectively. The cultured CPHs began to adhere to the wall at 4 h, and the medium was changed for the first time after 12 h. After that, the cells were treated differently according to specific needs. The separated CPPAs was basically completed adherence in 12 h. After 24 h, the cell state was relatively stable and the medium was changed to remove blood cells and other impurities. CPHs and CPPAs were cultured at 37 °C in a 5% CO_2_ humidified atmosphere.

### Plasmid construction and RNA interference

The plasmid pcDNA3.1-*GHR* was artificially synthesized by GeneCreate Biological Engineering (Wuhan, China), which were further confirmed by Sanger sequencing in Table S1. In addition, siRNAs used for the *GHR* knockdown were synthesized by Guangzhou RiboBio (Guangzhou, China). si-*GHR* sequence was 5′-CCUCGAUUUGGAUACCAUA-3′.

### Transfection

Cells were plated in different culture plates and incubated overnight before transfection. Then, pcDNA3.1-*GHR* and pcDNA3.1 as well as si-GHR and si-NC were transfected in cells using Lipofectamine 3000 reagent (Invitrogen, CA, USA) according to the manufacturer’s protocol. In brief, after waiting for the confluence of cells in the 12-well plate reached 60–80% with the better growth state and the uniform density, diluted 3 μL Lipofectamine 3000 liposomes with 50 μL serum-free opti-MEM culture medium, and mixed them gently with a pipette. Taken 50 μL serum-free opti-MEM culture medium to dilute the DNA or siRNA, then mixed 2 μL P3000 reagent with the above two mixtures gently. After incubated at room temperature for 10 min, added 100 μL of the incubated DNA-liposome mixture to each well. After 6 h transfection, observed the cell growth status under a microscope, and replaced the medium with fresh Williams’E or DMEM/F12 medium containing 15% fetal bovine serum. The cells were analyzed at 48 h after transfection.

### Detection of malondialdehyde level

Malondialdehyde (MDA) levels were detected using an MDA assay kit (S0131; Beyotime, Shanghai, China) according to the manufacturer’s protocol. Absorbance was determined by a Fluorescence/Multi-Detection Microplate Reader (BioTek, USA). Data were normalized to the control group and expressed as a percentage of control levels.

### Detection of adenosine triphosphate level

ATP levels were measured using an ATP assay kit (Beyotime, China) according to the manufacturer’s protocol. ATP levels were determined by a Fluorescence/Multi-Detection Microplate Reader (BioTek, USA). Data were normalized to the control group and are expressed as a percentage of control levels.

### Detection of mitochondrial membrane potential

ΔΨm was measured using a JC-1 kit (Beyotime, China) according to the manufacturer’s protocol. The mitochondria were fixed with JC-1 for 20 min at 37 °C. Fluorescence was determined by a Fluorescence/Multi-Detection Microplate Reader (BioTek, USA) as previous described [27]. Data (the ratio of aggregated and monomeric JC-1) were normalized to the control group and are expressed as a percentage of control levels.

### Statistical analyses

All experiments were performed at least three times. The data are presented as means ± standard error of the means. Statistical analyses were performed using Student’s *t*-test, and we took *P* < 0.05 to indicate statistical significance, *P < 0.05, **P < 0.01, ***P < 0.001.

## Supplementary Information

**Additional file 1: Table S1**. Sanger sequencing of pcDNA3.1-*GHR* by universal primers.

**Additional file 2: Figure S1**.

## Data Availability

All data generated or analyzed during this study are available from the corresponding author on reasonable request.

## Abbreviations

AMPK: AMP-activated protein kinase
ATP: Adenosine triphosphate
CPHs: Chicken primary hepatocytes
CPPAs: Chicken primary pre-adipocytes
GHR: Growth hormone receptor
MDA: Malondialdehyde
ΔΨm: mitochondrial membrane potential
OXPHOS: Oxidative phosphorylation
PCR: Polymerase chain reaction
PGC1α: Peroxisome proliferator-activated receptor γ co-activator 1α
qRT-PCR: Quantitative real-time PCR
SLD: Sex-linked dwarf; TG, triglyceride
