# Supplementary material for: Growth hormone receptor gene influences mitochondrial function and chicken lipid metabolism by AMPK-PGC1α-PPAR signaling pathway
Source: BMC Genomics. 2022 Mar 19;23:219. doi: 10.1186/s12864-021-08268-9 (PMC8933938; doi:10.1186/s12864-021-08268-9)
Supplement: Supplementary file 1 — Additional file 1: Table S1. Sanger sequencing of pcDNA3.1-GHR by universal primers. [file 12864_2021_8268_MOESM1_ESM.docx]

**Table S1** Sanger sequencing of pcDNA3.1-*GHR* by universal primers.

**Table S1** Sanger sequencing of pcDNA3.1-*GHR* by universal primers.

| Genes | Sanger sequencing | Notes |
| --- | --- | --- |
| *GHR* | atggatcttcggcatctgctgtttactttggcactggtgtgtgcaaatgactcactttctgcaagtgatgatcttctgcagtggccacaaatcagcaagtgcaggtcacctgagctggagacattttcgtgttattggactgatggaaaggtcactacttcaggaacaatacaactgttgtatatgaaaaggagtgatgaagactggaaagaatgtccggattatatcactgcaggagaaaatagctgttacttcaacacatcctacacctcgatttggataccatattgtgttaagcttgccaataaagatgaagtatttgacgaaaagtgtttcagtgttgatgaaatagtactacctgatccccctgtgcaccttaactggactctgctaaatactagtcaaactgggatccatggggatattcaagtacgatgggatccaccaccaacagcagatgttcagaaaggatggattactctggagtatgaattgcagtacaaagaagttaatgagacaaaatggaaggagttagaacccaggctctcaacagtggttccactgtattctctgaagatgggaagagattatgagatccgagtccgatcaagacaacgtacctccgaaaagtttggggagttcagtgaaatcctctatgtttcctttactcaagcaggcattgaatttgttcattgtgctgaagaaatcgagtttccctggttcttagttgttgtcttcggagtgtgtgggctggccgtaacagcgatcttaatcctgttgtctaaacagccaaggttaaaaatgctgatttttcctcctgtgccagttccaaagattaaagggattgacccagatctcttaaagaaaggaaagctagatgaagtgaactccatcttagccagccatgacaactacaagacacagctatacaatgatgacttgtgggttgagtttattgaattggacatagatgactccgatgaaaagaacagagtctcagatactgacaggctcctgagtgacgatcatctgaagtcacacagttgcttgggagccaaggatgatgattctggacgtgccagttgttatgaaccagatattccagagacagacttcagtgcaagcgacacatgtgatgccatctctgatattgatcagttcaagaaggtaactgaaaaagaagaggatctcttgtgccttcataggaaagatgatgttgaggcacttcaaagtcttgccaacacagatacccaacagccgcatactagtactcagtctgaaagcagagagtcatggccaccttttgcagacagcactgactcagctaatccatcagtccaaactcagctaagtaaccagaattccctgacaaacactgacttctatgctcaagtgagtgatattactcctgctggaagtgttgtactttctccagggcagaaatccaaggtgggaagagcacagtgtgaaagctgcacagaacaaaacttcaccatggacaatgcctatttctgtgaggcagatgtgaaaaaatgtattgctgtgatttcacaggaagaggatgagccgcgtgttcaggagcaaagctgtaacgaggacacttacttcaccacagaaagccttaccactaccggtatcaatcttggagcttcaatggcagaaaccccaagtatggaaatgcctgtcccagactacacttctattcatattgttcactctccacaaggccttgtgctcaatgcaactgcactgcctgtgccagagaaagaatttaacatgtcttgtggctatgtgagcacagaccagctgaacaaaatcatgccgtag | Verify of plasmid pcDNA3.1-GHR  construct |
