# Supplementary figures and images for: Growth hormone receptor gene influences mitochondrial function and chicken lipid metabolism by AMPK-PGC1α-PPAR signaling pathway
Source: BMC Genomics. 2022 Mar 19;23:219. doi: 10.1186/s12864-021-08268-9 (PMC8933938; doi:10.1186/s12864-021-08268-9)

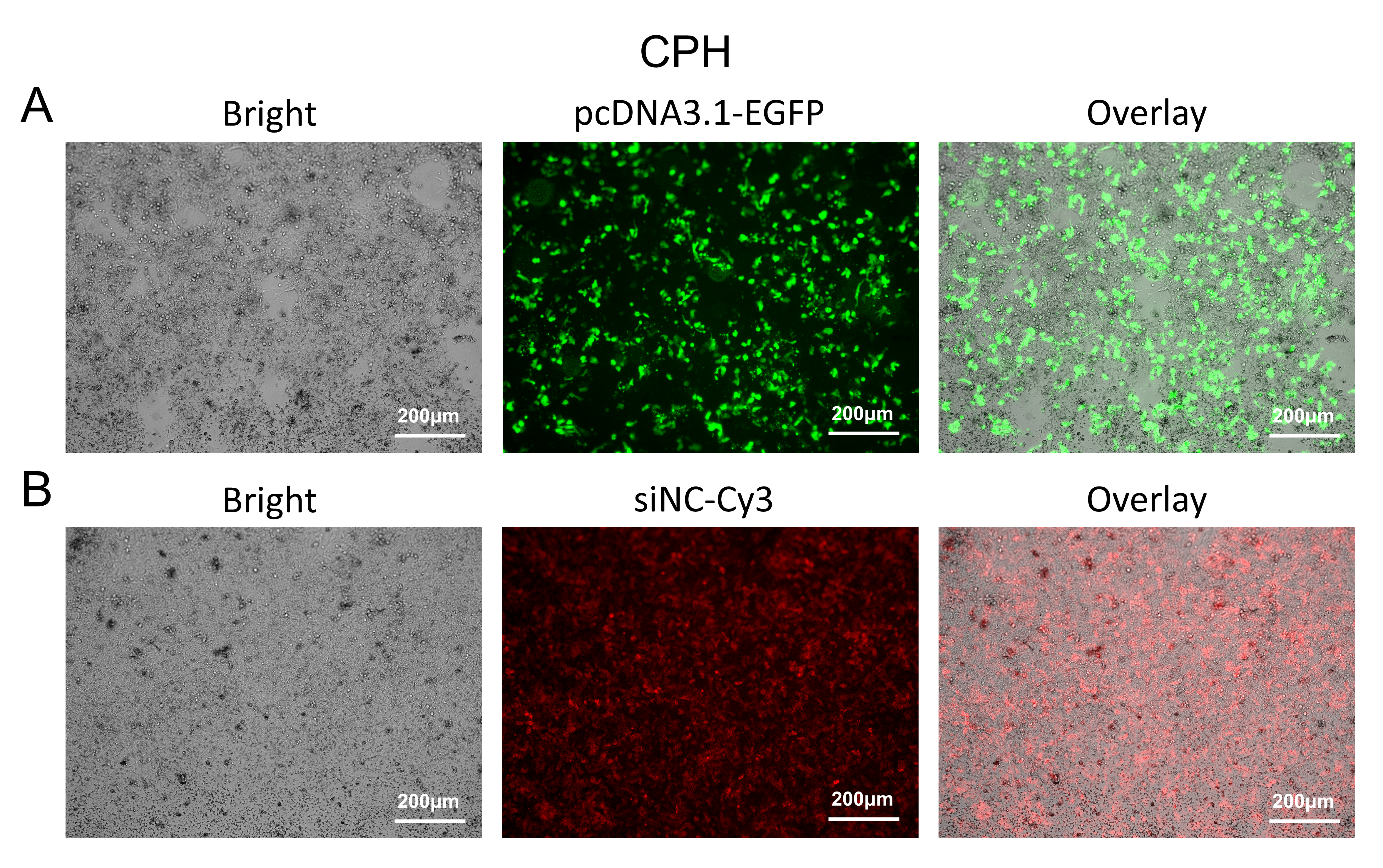

Supplement: Supplementary file 2 — Additional file 2: Figure S1. [file 12864_2021_8268_MOESM2_ESM.tif]
